# Association of heartbeat complexity with survival in advanced non-small cell lung cancer patients

**DOI:** 10.3389/fnins.2023.1113225

**Published:** 2023-04-12

**Authors:** Shuang Wu, Guangqiao Li, Man Chen, Sai Zhang, Yufu Zhou, Bo Shi, Xiaochun Zhang

**Affiliations:** ^1^School of Medicine, Yangzhou University, Yangzhou, Jiangsu, China; ^2^Department of Radiation Oncology, First Affiliated Hospital, Bengbu Medical College, Bengbu, Anhui, China; ^3^School of Medical Imaging, Bengbu Medical College, Bengbu, Anhui, China; ^4^Anhui Key Laboratory of Computational Medicine and Intelligent Health, Bengbu Medical College, Bengbu, Anhui, China; ^5^Department of Oncology, Yangzhou Hospital of Traditional Chinese Medicine, Yangzhou, Jiangsu, China

**Keywords:** heart rate variability, nonlinear methods, heartbeat complexity, recurrence quantification analysis, advanced non-small cell lung cancer, prognosis

## Abstract

**Background:**

Previous studies have shown that the predictive value of traditional linear (time domain and frequency domain) heart rate variability (HRV) for the survival of patients with advanced non-small cell lung cancer (NSCLC) is controversial. Nonlinear methods, based on the concept of complexity, have been used to evaluate HRV, providing a new means to reveal the physiological and pathological changes in HRV. This study aimed to assess the association between heartbeat complexity and overall survival in patients with advanced NSCLC.

**Methods:**

This study included 78 patients with advanced NSCLC (mean age: 62.0 ± 9.3 years). A 5-min resting electrocardiogram of advanced NSCLC patients was collected to analyze the following HRV parameters: time domain indicators, i.e., standard deviation of the normal-normal intervals (SDNN) and root mean square of successive interval differences (RMSSD); frequency domain indicators, i.e., total power (TP), low frequency power (LF), high frequency power (HF), and the ratio of LF to HF (LF/HF); nonlinear HRV indicators characterizing heartbeat complexity, i.e., approximate entropy (ApEn), sample entropy (SampEn), and recurrence quantification analysis (RQA) indexes: mean diagonal line length (Lmean), maximal diagonal line length (Lmax), recurrence rate (REC), determinism (DET), and shannon entropy (ShanEn).

**Results:**

Univariate analysis revealed that the linear frequency domain parameter HF and nonlinear RQA parameters Lmax, REC, and DET were significantly correlated with the survival of advanced NSCLC patients (all *p* < 0.05). After adjusting for confounders in the multivariate analysis, HF, REC, and DET were found to be independent prognostic factors for the survival of patients with advanced NSCLC (all *p* < 0.05).

**Conclusion:**

There was an independent association between heartbeat complexity and survival in advanced NSCLC patients. The nonlinear analysis method based on RQA may provide valuable additional information for the prognostic stratification of patients with advanced NSCLC and may supplement the traditional time domain and frequency domain analysis methods.

## Introduction

Lung cancer (LC) is the second most common cancer and the leading cause of cancer deaths worldwide (Sung et al., 2021). The pathological types of LC are mainly divided into non-small cell lung cancer (NSCLC; including squamous cell carcinoma and adenocarcinoma) and small cell lung cancer, of which NSCLC accounts for 85% of cases (Zheng, 2016). Previous studies have confirmed that abnormal increases in sympathetic activity or abnormal decreases in vagal activity (an important part of the parasympathetic nervous system) are related to the occurrence and development of LC (Wang H. M. et al., 2013; Gidron et al., 2018; Ha et al., 2019). Sympathetic nerves can promote tumor progression by regulating the inflammatory response (Huan et al., 2017) and angiogenesis (Garg et al., 2017), while vagus nerve activation can inhibit the inflammatory response (Tracey, 2009) and sympathetic nerve activity (Saku et al., 2014).

Heart rate variability (HRV) refers to the variation in time intervals between adjacent heartbeats and is considered a reliable indicator for the quantitative evaluation of autonomic nervous system activity (Camm et al., 1996; Lombardi and Stein, 2011). Traditional linear (time domain or frequency domain) HRV analysis is the main method to clinically evaluate autonomic function (Camm et al., 1996; Lombardi and Stein, 2011). Growing research has demonstrated the essential role of inflammatory cytokines, such as tumor necrosis factor-alpha (TNF-α), interleukin 6 (IL-6), and C-reactive protein (CRP), in promoting tumor occurrence and growth (Balkwill, 2009; Taniguchi and Karin, 2014; Hart et al., 2020). In a recent study, vagally mediated HRV parameters were inversely associated with levels of inflammatory markers such as TNF-α, IL-6, and CRP, possibly because vagal nerve stimulation reduces inflammatory cytokines through the cholinergic anti-inflammatory pathway (Williams et al., 2019). Published studies have suggested that linear (time domain or frequency domain) HRV is associated with the prognosis of patients with malignant tumors, including LC with brain metastasis (Wu et al., 2022), brain metastasis (Wang et al., 2021), gastric cancer (Hu et al., 2018), colorectal cancer (Mouton et al., 2012), liver cancer (Chiang et al., 2010), pancreatic cancer (De Couck et al., 2016), breast cancer (Giese-Davis et al., 2015), and so on. Although accumulating evidence suggests that linear HRV may be a prognostic marker for patients with malignant tumors, its predictive value in the survival of NSCLC patients remains controversial. For example, Kim et al. (2015) performed a univariate analysis, which showed that there was a significant correlation between the standard deviation of all normal-to-normal intervals (SDNN) and the overall survival of advanced NSCLC patients, while their multivariate analysis indicated that SDNN was not an independent prognostic factor for the overall survival of patients with advanced NSCLC. De Couck et al. (2013) confirmed that SDNN and the root mean square of successive interval differences (RMSSD) could not significantly predict the overall survival of NSCLC patients, while further analysis showed that they could significantly predict the overall survival of NSCLC patients who were under 65 years of age (all *p* < 0.05). This may be because the true effect of linear HRV analysis is greatly affected by the heterogeneity in the tumor stage of NSCLC patients and the length of ECG data used in the study (De Couck et al., 2013; Kim et al., 2015).

The human body is characterized by nonstationarity and nonlinearity, and simple linear information may be insufficient to correctly describe the complex nonlinear behavior that dominates the human system. The nonlinear analysis method accords with the nonlinear and nonstationary characteristics of heartbeat interval time series, providing a new perspective for revealing the physiopathological changes in HRV. It can not only reflect more information about heart rate dynamics but also complement traditional time domain and frequency domain analysis. The unpredictability or complexity of signals is one of the main characteristics of nonlinear heart rate dynamics. A healthy human system exhibits spatial and temporal complexity, but disease may involve an increase or decrease in complexity (Vaillancourt and Newell, 2002). Previous studies have also shown that compared with healthy people, the heartbeat complexity of patients with atrial fibrillation (Mohebbi and Ghassemian, 2011), coronary artery disease (Acharya et al., 2014), or acute myocardial ischemia (Peng and Sun, 2011) is significantly lower. Recently, some studies have been conducted to preliminarily examine the prognostic value of nonlinear HRV parameters in cancer (Shi et al., 2019; Escutia-Reyes et al., 2021; Li et al., 2022). For example, Shi et al. (2019) found that decreased heartbeat complexity was associated with higher carcinoembryonic antigen levels in gastric cancer patients. Li et al. (2022) showed that lower heartbeat complexity was predictive of shorter survival of LC patients with brain metastasis. However, few studies have explored the role of heartbeat complexity in the prognosis of patients with advanced NSCLC.

At present, it is not clear whether there is a correlation between heartbeat complexity and prognosis in advanced NSCLC patients. Therefore, this study aimed to verify the relationship between heartbeat complexity and overall survival in patients with advanced NSCLC.

## Methods

### Subjects

This prospective study enrolled NSCLC patients who were treated in the hospital from October 2019 to February 2021, with the approval of the Medical Ethics Committee of the First Affiliated Hospital of Bengbu Medical College. The inclusion criteria were as follows: (1) NSCLC confirmed by pathological examination and (2) stage III and IV NSCLC. The exclusion criteria were as follows: (1) installation of a cardiac pacemaker, (2) use of antiarrhythmic drugs or β-blockers, (3) complications with other types of malignant tumors, (4) lack of clinical or pathological data, and (5) treatment with chemotherapy, radiotherapy, or surgery within 3 weeks before data collection. The research process was carried out in accordance with the Helsinki Declaration. All patients provided written informed consent before study enrolment.

### Data collection

The medical staff informed patients about the study and used a single-lead Micro-ECG recorder (HeaLink-R211B; HeaLinkLtd., Bengbu, China) to collect 5 min of ECG data from NSCLC patients at a 400 Hz sampling rate and V6-lead in an undisturbed quiet room. The participants, fully relaxed and in the supine position, were asked to breathe regularly and gently and could neither speak nor move their bodies during the measurement.

We collected the following clinical background information from advanced NSCLC patients: sex, age, body mass index (BMI), smoking history, Karnofsky performance status (KPS), pathological type, prior treatment history (radiotherapy, chemotherapy, targeted therapy, and surgery), TNM stage, and overall survival. Patient overall survival was defined from the date of HRV detection to the date of death or the last follow-up. The patients were followed up by telephone call backs or by consulting the case data, and the last follow-up date was on September 03, 2022.

### HRV analysis

This research used the Pan-Tompkins algorithm to extract R-R interval time series on ECG and to calculate linear (time domain or frequency domain) and nonlinear HRV parameters (Pan and Tompkins, 1985). An automatic artifact correction algorithm in the Kubios software was used to correct technical and physiological artifacts within R-R intervals. The estimated values for respiratory rate (RR) were calculated using an ECG-derived respiratory method (Moody et al., 1985). The following common time domain and frequency domain parameters were used: SDNN, RMSSD, total power (TP, 0–0.4 Hz), high frequency power (HF, 0.15–0.4 Hz), low frequency power (LF, 0.04–0.15 Hz), and the ratio of LF to HF (LF/HF). The power spectrum signal was analyzed in the frequency domain, and the fast Fourier transform algorithm was used to calculate the power spectrum density (Camm et al., 1996; Vanderlei et al., 2009; Lombardi and Stein, 2011).

Through the analysis of HRV by nonlinear dynamics, the following indexes characterizing the complexity of heartbeat were obtained [i.e., approximate entropy (ApEn), sample entropy (SampEn), and recurrence quantification analysis (RQA): mean diagonal line length (Lmean), maximal diagonal line length (Lmax), recurrence rate (REC), determinism (DET), and shannon entropy (ShanEn)]. ApEn and SampEn are typical nonlinear dynamic methods for quantifying the complexity and regularity of time series. The greater the complexity and randomness of the time series, the greater their values (Voss et al., 2009; de Godoy, 2016). SampEn aims to provide better consistency than ApEn (Yentes et al., 2013). RQA is a quantitative description of the deterministic structure and complexity in recursive graphs that reveals the system dynamical behavior. The smaller the Lmean and Lmax are, the higher the complexity and instability of the system. The higher the REC value is, the stronger the similarity of system dynamics. DET is an index used to quantify the regularity and certainty of system dynamics, and the higher the DET value is, the stronger the certainty. ShanEn is a measure of signal complexity. The smaller the ShanEn value, the closer it is to a chaotic dynamic behavior (Marwan et al., 2002, 2007; Sun and Wang, 2008).

The parameters for ApEn and SampEn were set to the embedding dimension *m* = 2, delay time τ = 1 and the tolerance value *r* = 0.2 SD (SD is the standard deviation; Burioka et al., 2005; Lee et al., 2013; Mohseni et al., 2022). The parameters of RQA were set to the embedding dimension *m* = 10, delay time τ = 1 and distance threshold *r* = √m SD (SD is the standard deviation of the R-R time series; Webber and Zbilut, 1994; Dabiré et al., 1998; Zimatore et al., 2017). Linear and nonlinear HRV indicators were analyzed by Kubios HRV Premium software (version 3.1.0, https://www.kubios.com Magi Kubios Oy, Kuopio, Finland; Niskanen et al., 2004).

### Statistical analysis

The sample size was estimated based on a previously published study about the association between vagal neuroimmunomodulation and the NSCLC survival rate (Gidron et al., 2018), and no specific statistical method was used to determine sample size. We added approximately 10% (*n* = 78) based on the Gidron et al. (2018) sample size (*n* = 71) in NSCLC patients. The normal continuous data are described as *x* ± *s*, the nonnormal continuous data are described as M [Q1 and Q3], and the counting data are described as the frequency and percentage. Univariate Cox regression analysis was performed to determine the significant prognostic factors between the included clinical factors and the additional factors affecting HRV [mean heart rate (mean HR), RR]. The optimal cut-off value for the HRV parameters for evaluating the overall survival of patients was obtained by X-Tile software (Camp et al., 2004). The event-survival curve was constructed by the Kaplan–Meier method to estimate the median overall survival. Finally, considering the correlation among HRV indices, we performed a multivariate Cox regression analysis for each HRV indicator individually with the prognostic confounding factors that were shown to be significant in the univariate analysis to evaluate the independent prognostic HRV parameters affecting NSCLC patients. All the data were analyzed by SPSS Statistics 25.0 (IBM Corp., Chicago, Illinois, United States). All analyses were two-tailed tests, and a *p* value of <0.05 was considered statistically significant.

## Results

Table 1 shows the general characteristics and HRV parameters of the advanced NSCLC patients. A total of 78 patients diagnosed with advanced NSCLC were included in this study, including 21 females and 57 males. The mean age was 62.0 ± 9.3 years. Forty-seven patients (60.3%) died, and 31 patients (39.7%) survived. The range of follow-up time was 0.5–34.8 months, with a median follow-up time of 21.4 months.

**Table 1:** Basic characteristics of the non-small cell lung cancer patients enrolled.

Characteristics	Values (*N* = 78)
Sex
Female	21 (26.9%)
Male	57 (73.1%)
Age (year)	62.0 ± 9.3
BMI (kg/m^2^)	23.8 ± 3.4
Mean HR (bpm)	81.5 ± 13.7
RR (Hz)	0.32 ± 0.08
Smoking
No	60 (76.9%)
Yes	18 (23.1%)
KPS
≤70	15 (19.2%)
>70	63 (80.8%)
Pathological types
Squamous cell carcinoma	18 (23.1%)
Adenocarcinoma	56 (71.8%)
Others	4 (5.1%)
Radiotherapy
Without	23 (29.5%)
With	55 (70.5%)
Chemotherapy
Without	16 (20.5%)
With	62 (79.5%)
Targeted therapy
Without	34 (43.6%)
With	44 (56.4%)
Surgery
Without	62 (79.5%)
With	16 (20.5%)
TNM stage
IIIA	10 (12.8%)
IIIB	8 (10.3%)
IIIC	4 (5.1%)
IVA	34 (43.6%)
IVB	22 (28.2%)
SDNN (ms)	18.9 [11.6, 25.2]
RMSSD (ms)	10.2 [5.8, 16.1]
TP (ms^2^)	204 [112, 467]
LF (ms^2^)	37 [19, 82]
HF (ms^2^)	33 [8, 70]
LF/HF	1.360 [0.554, 3.645]
ApEn	1.101 [1.022, 1.171]
SampEn	1.339 ± 0.376
Lmean (beats)	15.93 [12.66, 23.58]
Lmax (beats)	325.0 [192.3, 401.0]
REC (%)	43.83 [34.05, 48.63]
DET (%)	99.24 [98.14, 99.56]
ShanEn	3.578 ± 0.461

Values are expressed as the number of patients (percentage) or mean ± SD or median [Q1, Q3].

BMI, body mass index; Mean HR, mean heart rate; bpm, beats per minute; RR, respiration rate; KPS, Karnofsky performance status; TNM, tumor-node-metastasis; SDNN, standard deviation of all normal-to-normal intervals; RMSSD, root mean square of successive differences; TP, total power; LF, low-frequency power; HF, high-frequency power; LF/HF, ratio of low-frequency power to high-frequency power; ApEn, approximate entropy; SampEn, sample entropy; Lmean, mean diagonal line length; Lmax, maximal diagonal line length; REC, recurrence rate; DET, determinism; ShanEn, Shannon entropy; SD, standard deviation; Q1, first quartile; and Q3, third quartile.

Univariate analysis showed that KPS and surgical history were significantly correlated with the overall survival of advanced NSCLC patients [KPS: hazard ratio = 2.430, 95% confidence interval (CI): 1.275–4.631, *p* = 0.007; surgery: hazard ratio = 3.861, 95% CI: 1.383–10.778, *p* = 0.010]. In univariate analysis, the overall survival of patients with advanced NSCLC was not significantly associated with sex, age, BMI, mean HR, RR, smoking history, pathological type, radiotherapy history, chemotherapy history, targeted therapy history, or TNM stage (Table 2).

**Table 2:** Univariate Cox regression analysis of clinical characteristics and survival in non-small cell lung cancer patients.

	Univariate analysis	
	Hazard ratio (95% CI)	*p* value
Sex		0.238
Female	0.665 (0.338, 1.309)	
Male	Ref	
Age (year)	0.990 (0.959, 1.021)	0.509
BMI (kg/m^2^)	0.939 (0.860, 1.027)	0.168
Mean HR (bpm)	1.003 (0.982, 1.025)	0.782
RR (Hz)	16.451 (0.318, 851.677)	0.164
Smoking		0.833
No	1.078 (0.535, 2.171)	
Yes	Ref	
KPS		**0.007**
≤ 70	2.430 (1.275, 4.631)	
> 70	Ref	
Pathological types		0.752
Squamous cell carcinoma	1.753 (0.388, 7.917)	
Adenocarcinoma	1.510 (0.363, 6.291)	
Others	Ref	
Radiotherapy		0.157
Without	0.622 (0.322, 1.201)	
With	Ref	
Chemotherapy		0.460
Without	1.291 (0.656, 2.537)	
With	Ref	
Targeted therapy		0.134
Without	0.629 (0.343, 1.154)	
With	Ref	
Surgery		**0.010**
Without	3.861 (1.383, 10.778)	
With	Ref	
TNM Stage		0.074
IIIA	0.404 (0.150, 1.087)	
IIIB	0.453 (0.153, 1.340)	
IIIC	0.654 (0.193, 2.216)	
IVA	0.399 (0.204, 0.781)	
IVB	Ref	

Bold *p* values indicate statistical significance (*p* value < 0.05).

CI, confidence interval; BMI, body mass index; Mean HR, mean heart rate; bpm, beats per minute; RR, respiration rate; KPS, Karnofsky performance status; and TNM, tumor-node-metastasis.

**Figure 1:**
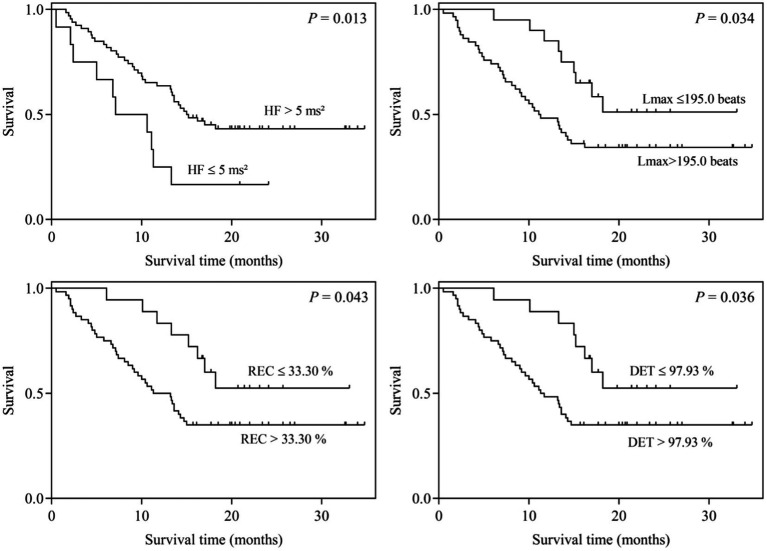
Kaplan–Meier survival curves for patients stratified by HF, Lmax, REC, and DET.

Univariate and multivariate analyses were performed to determine the correlation between heartbeat complexity and survival in patients with advanced NSCLC. Univariate analysis showed that there were significant correlations between the frequency domain parameter HF as well as the RQA indicators Lmax, REC, and DET and the overall survival of advanced NSCLC patients. Specifically, compared with the high-value HF group, the low-value HF group had a poorer prognosis (7.1 vs. 15.0 months, *p* = 0.016). Compared with those in the low-value Lmax, REC, and DET group, the NSCLC patients in the high-value Lmax, REC, and DET group had a poorer prognosis (Lmax: 17.1 vs. 23.6 months, *p* = 0.039; REC: 17.3 vs. 23.9 months, *p* = 0.048; and DET: 17.2 vs. 24.1 months, *p* = 0.041; Table 3; Figure 1).

**Table 3:** Univariate and multivariate analyses of HRV variables as predictors of survival.

	Median survival (M)	Univariate analysis		Multivariate analysis	
		Hazard ratio (95% CI)	*p* value	Hazard ratio (95% CI)	*p* value
SDNN (ms)
≤ 23.5	13.6	1.412 (0.718, 2.775)	0.317	1.343 (0.680, 2.654)	0.396
> 23.5	16.2	Ref		Ref	
RMSSD (ms)
≤ 8.9	11.3	1.413 (0.797, 2.506)	0.237	1.664 (0.911, 3.041)	0.098
> 8.9	16.2	Ref		Ref	
TP (ms^2^)
≤ 67	13.3	1.502 (0.672, 3.360)	0.321	1.121 (0.485, 2.587)	0.790
> 67	14.3	Ref		Ref	
LF (ms^2^)
≤ 31	11.1	1.563 (0.880, 2.775)	0.127	1.495 (0.841, 2.657)	0.170
> 31	16.2	Ref		Ref	
HF (ms^2^)
≤ 5	7.1	2.382 (1.174, 4.830)	**0.016**	2.108 (1.035, 4.291)	**0.040**
> 5	15.0	Ref		Ref	
LF/HF
≤ 4.950	14.7	0.553 (0.266, 1.146)	0.111	0.525 (0.252, 1.094)	0.085
> 4.950	9.6	Ref		Ref	
ApEn
≤ 1.140	14.7	0.703 (0.394, 1.254)	0.232	0.711 (0.396, 1.278)	0.254
> 1.140	11.3	Ref		Ref	
SampEn
≤ 1.620	17.7	2.114 (0.896, 4.989)	0.087	1.784 (0.750, 4.247)	0.191
> 1.620	24.1	Ref		Ref	
Lmean (beats)
≤ 10.74	18.2	0.528 (0.236, 1.180)	0.120	0.571 (0.252, 1.294)	0.180
> 10.74	13.3	Ref		Ref	
Lmax (beats)
≤ 195.0	23.6	0.464 (0.224, 0.961)	**0.039**	0.490 (0.236, 1.017)	0.056
> 195.0	17.1	Ref		Ref	
REC (%)
≤ 33.30	23.9	0.463 (0.216, 0.993)	**0.048**	0.415 (0.192, 0.896)	**0.025**
> 33.30	17.3	Ref		Ref	
DET (%)
≤ 97.93	24.1	0.450 (0.210, 0.967)	**0.041**	0.432 (0.200, 0.935)	**0.033**
> 97.93	17.2	Ref		Ref	
ShanEn
≤ 3.121	18.2	0.528 (0.236, 1.180)	0.120	0.571 (0.252, 1.294)	0.180
> 3.121	13.3	Ref		Ref	

Bold *p* values indicate statistical significance (*p* value < 0.05).

CI, confidence interval; SDNN, standard deviation of all normal-to-normal intervals; RMSSD, root mean square of successive differences; TP, total power; LF, low-frequency power; HF, high-frequency power; LF/HF, ratio of low-frequency power to high-frequency power; ApEn, approximate entropy; SampEn, sample entropy; Lmean, mean diagonal line length; Lmax, maximal diagonal line length; REC, recurrence rate; DET, determinism; and ShanEn, Shannon entropy.

In multivariate analysis, the frequency domain parameter HF and RQA indexes REC and DET were still shown to be important prognostic factors for the overall survival of patients with advanced NSCLC (HF: *p* = 0.040, hazard ratio = 2.108, 95% CI: 1.035–4.291; REC: *p* = 0.025, hazard ratio = 0.415, 95% CI: 0.192–0.896; DET: *p* = 0.033, hazard ratio = 0.432, 95% CI: 0.200–0.935). There was no significant correlation between Lmax and the overall survival of advanced NSCLC patients (*p* = 0.056, hazard ratio = 0.490, 95% CI: 0.236–1.017; Table 3).

## Discussion

This study verified the correlation between heartbeat complexity and overall survival in patients with advanced NSCLC. Our study showed that the frequency domain parameter HF and RQA indexes Lmax, REC, and DET were significantly correlated with the overall survival of advanced NSCLC patients. In multivariate analysis, the frequency domain parameters HF and RQA indexes REC and DET were independently correlated with the overall survival of advanced NSCLC patients after adjusting for confounders.

The autonomic nervous system includes the sympathetic nervous system and the parasympathetic nervous system, which can antagonize or cooperate to maintain the normal physiological activities of the body (Schwartz and De Ferrari, 2011). The sympathetic nerve can regulate the pathological process of tumor growth (Coelho et al., 2017) and metastasis (Sloan et al., 2010), and the vagus nerve can affect tumor progression through systemic anti-inflammatory pathways (Hajiasgharzadeh et al., 2019) and the inhibition of sympathetic activity (Saku et al., 2014). At present, there are a variety of detection techniques to reflect autonomic function, among which HRV is a non-invasive and easily accessible detection method that has been used in clinical settings. It provides effective help for understanding the relationship between autonomic function and prognostic information in patients with malignant tumors (De Couck et al., 2018; Kloter et al., 2018). Previous studies revealed that there is a significant positive correlation between higher vagal activity and better prognosis in NSCLC patients (De Couck et al., 2013; Gidron et al., 2018). For example, Gidron et al. (2018) confirmed the relationship between neuroimmunomodulation (i.e., the ratio of RMSSD to CRP) and the NSCLC survival rate. The results showed that compared with those in the lower neuroimmunomodulation group, the NSCLC patients in the higher neuroimmunomodulation group had a better prognosis (475.2 vs. 285.1 days, *p* < 0.05). Therefore, Gidron et al. (2018) proposed that vagal regulation of inflammation may be a new biomarker for the prognosis of patients with NSCLC.

Time domain analysis is the simplest and easiest method to study HRV and can provide relatively clear physiological information, which is easily accepted by clinicians. The published literature suggests that SDNN and RMSSD are significantly associated with the overall survival of patients with advanced malignant tumors (Wang Y. M. et al., 2013; De Couck et al., 2016; Wu et al., 2022). For example, Wang Y. M. et al. (2013) showed that SDNN is an independent prognostic factor for the overall survival of patients with brain metastasis. De Couck et al. (2016) found that SDNN can significantly predict the overall survival in patients with advanced pancreatic cancer. In addition, our previous study showed that there is an independent association between RMSSD and overall survival in LC patients with brain metastasis (Wu et al., 2022). Although most previous studies showed that vagal activity based on linear HRV has a prognostic effect on cancer patients, a few studies found that the change in linear HRV is not an appropriate marker for predicting overall survival in advanced NSCLC patients. For instance, Kim et al. (2015) found that although there was a significant difference in the median survival between advanced NSCLC patients with SDNN ≥ 20.0 ms and those with SDNN < 20.0 ms (213 vs. 155 days, *p* = 0.029), SDNN was not an independent prognostic factor for overall survival in patients with advanced NSCLC. Similar to the study of Kim et al. (2015), our study found that compared with advanced NSCLC patients in the low-value SDNN or RMSSD group, patients in the high-value SDNN or RMSSD group had a better prognosis (SDNN: 13.6 vs. 16.2 months; RMSSD: 11.3 vs. 16.2 months). However, SDNN and RMSSD were not independent prognostic factors for the overall survival of advanced NSCLC patients. This may be because the 22 patients with stage III NSCLC in this study received more active antitumor therapy (such as radiotherapy or surgery). While antitumor therapy successfully reduces the tumor load, it may mask the effect of autonomic function on patient prognosis to some extent, which might weaken the evaluation effect of the autonomic nervous system (investigated by linear HRV parameters; De Couck et al., 2018). Some studies have been conducted to preliminarily investigate the short-term effect of different antitumor treatments on linear HRV parameters (Hoca et al., 2012; Hansen et al., 2013; Stachowiak et al., 2018). It is not clear whether these treatments have short-term or long-term effects on nonlinear HRV parameters.

Frequency domain analysis is not only easy to implement but also a relatively accurate method to measure the regulatory state of the autonomic nervous system. Previous studies have indicated that the higher frequency domain parameter HF in patients with recurrent or metastatic breast cancer is closely related to their longer overall survival (Giese-Davis et al., 2015). Chiang et al. (2013) showed that there was a significant correlation between the frequency domain parameter HF of cancer patients without lung cancer and their 7-day survival rate. In addition, another study by Chiang et al. (2010) found that HF was significantly associated with prognosis in patients with advanced hepatocellular carcinoma. Similar to their results, the univariate analysis in our study revealed that there was a significant correlation between HF and the overall survival of advanced NSCLC patients (*p* = 0.016). In multivariate analysis, the correlation between overall survival and HF in patients with advanced NSCLC remained significant (*p* = 0.040, hazard ratio = 2.108, 95% CI: 1.035–4.291). HF reflects vagal activity (Vanderlei et al., 2009; Lombardi and Stein, 2011), indicating that vagal tone has a certain predictive value in the overall survival of patients with advanced NSCLC.

It is worth noting that most of the published studies use classical linear indexes (time domain or frequency domain) based on HRV analysis, reflecting the regulation of heart rate by sympathetic and parasympathetic nerves (De Couck et al., 2018; Kloter et al., 2018). Previous studies have also preliminarily explored the relationship between several nonlinear HRV parameters and the prognostic information of patients with malignant tumors (Bettermann et al., 2001; Shi et al., 2019; Escutia-Reyes et al., 2021; Wu et al., 2021). For example, Shi et al. (2019) found that the increase in irregularity and the decrease in complexity of heartbeat time series are significantly correlated with higher carcinoembryonic antigen levels in patients with gastric cancer. Bettermann et al. (2001) showed that compared with breast cancer patients without metastasis, the heartbeat complexity (reflected by ApEn) of breast cancer patients with metastasis was clearly lower. Escutia-Reyes et al. (2021) showed that nonlinear HRV parameters, such as the SampEn, were significantly different between a breast cancer survivor group and a cancer-free female control group. In our previous study exploring the correlation between short-term HRV and TNM staging in patients with breast cancer, the results showed that the nonlinear HRV parameters ApEn and SampEn, which characterize heartbeat complexity, were not significantly different between tumor stages (Wu et al., 2021). In this study, there was no significant correlation between ApEn or SampEn and the overall survival of advanced NSCLC patients (*p* > 0.05). Therefore, we strongly recommend that more background variables, such as type of cancer or severity of disease, and more entropy analysis parameters, such as distribution entropy, fuzzy entropy, permutation entropy, or multiscale entropy, can be included in future research to obtain more valuable prognostic information.

Because the RQA can be applied to short, nonstationary and high-noise signal sequences of the R-R interval, where there is not a strict requirement for the length of data, it has been used in many studies on heart rate dynamics (Mohebbi and Ghassemian, 2011; Acharya et al., 2014; Li et al., 2022). For example, Acharya et al. (2014) showed that Lmean, Lmax, REC, and DET in patients with coronary artery disease are significantly higher than those in healthy populations. Mohebbi and Ghassemian (2011) showed that compared with the ECG signals that are distant from paroxysmal atrial fibrillation, RQA indexes, such as Lmean, Lmax, REC, and ShanEn with the ECG signals that are before paroxysmal atrial fibrillation were significantly higher. Li et al. (2022) showed that higher Lmax is significantly associated with shorter overall survival in LC patients with brain metastasis. Similar to the above studies, our results showed that the RQA indexes REC and DET are independent prognostic factors for the overall survival of advanced NSCLC patients (*p* < 0.05), indicating that the increase in REC and DET, that is, the decrease in heartbeat complexity, is an independent risk factor for poor prognosis in patients with advanced NSCLC. In addition, in our study, compared with the low-value Lmax group, advanced NSCLC patients in the high-value Lmax group had a poorer prognosis (17.1 vs. 23.6 months). However, Lmax only tended to be significantly correlated with overall survival after adjusting for confounders (*p* = 0.056, hazard ratio = 0.490, 95% CI: 0.236–1.017), which may be related to the relatively small sample size in this study. Therefore, the results of the current research still needs to be confirmed with a long-term follow-up prospective study with further expansion of the sample size. The results of this study may provide new evidence for the role of heartbeat complexity in the prognosis of cancer patients. However, exact physiopathological mechanisms of nonlinear HRV parameters have yet to be fully elucidated in the prognosis of cancer patients. Vagally mediated linear HRV indicators were correlated with levels of inflammatory markers (Williams et al., 2019). We suggest to compare correlations between linear and nonlinear HRV parameters with inflammation markers in future studies, and this may provide some hints as to the better prognostic value of nonlinear HRV indicators in cancer patients.

## Limitations

One major limitation of our study is the heterogeneity of antitumor therapy modalities. All patients with stage III or IV NSCLC were enrolled in this study, and they received different antitumor treatments (such as radiotherapy or surgery). It is not clear whether these treatments have short-term or long-term effects on heartbeat complexity. Second, our sample size was too small to allow sufficiently powered statistical analysis to be performed. With a small sample size, the performance of too many statistical tests without any correction of the *p*-level maybe causes risking a type-1 error. Could it be that other issues may have led to more prognostic power seen in the nonlinear HRV parameters such as type of cancer or severity of disease, etc. factors that may affect more the linear HRV parameters and possibly less the nonlinear HRV parameters? More studies are needed to prove this speculation. Therefore, we strongly recommend expanding the sample size to conduct additional research on these differences.

## Conclusion

This study reveals that the RQA parameters REC and DET, which characterize heartbeat complexity, are independently related to the overall survival of patients with advanced NSCLC. This finding indicates that heartbeat complexity based on RQA may be used as a new prognostic indicator for advanced NSCLC patients and may complement the traditional time domain and frequency domain indicators.

## Data availability statement

The raw data supporting the conclusions of this article will be made available by the authors, without undue reservation.

## Ethics statement

The studies involving human participants were reviewed and approved by the Medical Ethics Committee of the First Affiliated Hospital of Bengbu Medical College. The patients/participants provided their written informed consent to participate in this study.

## Author contributions

BS: conceptualization, methodology, resources, and writing–review and editing. XZ and YZ: supervision and resources. SW: data collection, data analysis, and writing-original draft preparation. GL: data analysis and writing-original draft preparation. MC: data collection and writing-original draft preparation. SZ: writing-original draft preparation. All authors contributed to the article and approved the submitted version.

## Funding

This research was funded by the “512” Outstanding Talents Fostering Project of Bengbu Medical College (grant number BY51201312), the Natural Science Research Project of Bengbu Medical College (grant number 2020byzd013), and the Scientific Research Innovation Project of Bengbu Medical College (grant number BYKC201905).

## Conflict of interest

An immediate family member of BS owns stock in HeaLink Ltd., Bengbu, China.

The remaining authors declare that the research was conducted in the absence of any commercial or financial relationships that could be construed as a potential conflict of interest.

## Publisher’s note

All claims expressed in this article are solely those of the authors and do not necessarily represent those of their affiliated organizations, or those of the publisher, the editors and the reviewers. Any product that may be evaluated in this article, or claim that may be made by its manufacturer, is not guaranteed or endorsed by the publisher.

## References

[ref1] Acharya U. R. Faust O. Sree V. Swapna G. Martis R. J. Kadri N. A.. (2014). Linear and nonlinear analysis of normal and CAD-affected heart rate signals. Comput. Methods Prog. Biomed. 113, 55–68. doi: 10.1016/j.cmpb.2013.08.017, PMID: 24119391

[ref2] Balkwill F. (2009). Tumor necrosis factor and cancer. Nat. Rev. Cancer 9, 361–371. doi: 10.1038/nrc2628 19343034

[ref3] Bettermann H. Kröz M. Girke M. Heckmann C. (2001). Heart rate dynamics and cardiorespiratory coordination in diabetic and breast cancer patients. Clin. Physiol. 21, 411–420. doi: 10.1046/j.1365-2281.2001.00342.x, PMID: 11442574

[ref4] Burioka N. Miyata M. Cornélissen G. Halberg F. Takeshima T. Kaplan D. T.. (2005). Approximate entropy in the electroencephalogram during wake and sleep. Clin. EEG Neurosci. 36, 21–24. doi: 10.1177/155005940503600106, PMID: 15683194 PMC2563806

[ref5] Camm A. J. Malik M. Bigger J. T. Breithardt G. Cerutti S. Cohen R. J.. (1996). Heart rate variability: standards of measurement, physiological interpretation and clinical use. Task force of the European society of cardiology and the north American society of pacing and electrophysiology. Circulation 93, 1043–1065. doi: 10.1161/01.CIR.93.5.1043 8598068

[ref6] Camp R. L. Dolled-Filhart M. Rimm D. L. (2004). X-tile: a new bio-informatics tool for biomarker assessment and outcome-based cut-point optimization. Clin. Cancer Res. 10, 7252–7259. doi: 10.1158/1078-0432, PMID: 15534099

[ref7] Chiang J. K. Koo M. Kuo T. B. J. Fu C. H. (2010). Association between cardiovascular autonomic functions and time to death in patients with terminal hepatocellular carcinoma. J. Pain Symptom Manag. 39, 673–679. doi: 10.1016/j.jpainsymman.2009.09.014, PMID: 20413055

[ref8] Chiang J. K. Kuo T. B. J. Fu C. H. Koo M. (2013). Predicting 7-day survival using heart rate variability in hospice patients with non-lung cancers. PLoS One 8:e69482. doi: 10.1371/journal.pone.0069482, PMID: 23936027 PMC3720672

[ref9] Coelho M. Soares-Silva C. Brandão D. Marino F. Cosentino M. Ribeiro L. (2017). β-Adrenergic modulation of cancer cell proliferation: available evidence and clinical perspectives. J. Cancer Res. Clin. Oncol. 143, 275–291. doi: 10.1007/s00432-016-2278-1, PMID: 27709364 PMC11819197

[ref10] Dabiré H. Mestivier D. Jarnet J. Safar M. E. Chau N. P. (1998). Quantification of sympathetic and parasympathetic tones by nonlinear indexes in normotensive rats. Am. J. Phys. 275, H1290–H1297. doi: 10.1152/ajpheart.1998.275.4.H1290, PMID: 9746478

[ref11] De Couck M. Caers R. Spiegel D. Gidron Y. (2018). The role of the Vagus nerve in cancer prognosis: a systematic and a comprehensive review. J. Oncol. 2018, 1236787–1236711. doi: 10.1155/2018/1236787, PMID: 30057605 PMC6051067

[ref12] De Couck M. Maréchal R. Moorthamers S. Van Laethem J. L. Gidron Y. (2016). Vagal nerve activity predicts overall survival in metastatic pancreatic cancer, mediated by inflammation. Cancer Epidemiol. 40, 47–51. doi: 10.1016/j.canep.2015.11.007, PMID: 26618335

[ref13] de Couck M. A. R. I. J. K. E. van Brummelen D. Schallier D. de Grève J. A. C. Q. U. E. S. Gidron Y. (2013). The relationship between vagal nerve activity and clinical outcomes in prostate and non-small cell lung cancer patients. Oncol. Rep. 30, 2435–2441. doi: 10.3892/or.2013.2725, PMID: 24026706

[ref14] de Godoy M. F. (2016). Nonlinear analysis of heart rate variability: a comprehensive review. J. Cardiol. Ther. 3, 528–533. doi: 10.17554/j.issn.2309-6861.2016.03.101-4

[ref15] Escutia-Reyes D. de Jesús Garduño-García J. Emilio-López-Chávez G. Gómez-Villanueva Á. Pliego-Carrillo A. C. Soto-Piña A. E.. (2021). Differences in heart rate variability and body composition in breast cancer survivors and women without cancer. Sci. Rep. 11:14460. doi: 10.1038/s41598-021-93713-8, PMID: 34262078 PMC8280116

[ref16] Garg J. Feng Y. X. Jansen S. R. Friedrich J. Lezoualc'h F. Schmidt M.. (2017). Catecholamines facilitate VEGF-dependent angiogenesis via β2-adrenoceptor-induced Epac1 and PKA activation. Oncotarget 8, 44732–44748. doi: 10.18632/oncotarget.17267, PMID: 28512254 PMC5546514

[ref17] Gidron Y. De Couck M. Schallier D. De Greve J. Van Laethem J. L. Maréchal R. (2018). The relationship between a new biomarker of vagal Neuroimmunomodulation and survival in two fatal cancers. J Immunol Res 2018, 1–5. doi: 10.1155/2018/4874193, PMID: 29854838 PMC5964597

[ref18] Giese-Davis J. Wilhelm F. H. Tamagawa R. Palesh O. Neri E. Taylor C. B.. (2015). Higher vagal activity as related to survival in patients with advanced breast cancer: an analysis of autonomic dysregulation. Psychosom. Med. 77, 346–355. doi: 10.1097/PSY.0000000000000167, PMID: 25886831 PMC5509754

[ref19] Ha D. Malhotra A. Ries A. L. O'Neal W. T. Fuster M. M. (2019). Heart rate variability and heart rate recovery in lung cancer survivors eligible for long-term cure. Respir. Physiol. Neurobiol. 269:103264. doi: 10.1016/j.resp.2019.103264, PMID: 31376471 PMC6759398

[ref20] Hajiasgharzadeh K. Sadigh-Eteghad S. Mansoori B. Mokhtarzadeh A. Shanehbandi D. Doustvandi M. A.. (2019). Alpha7 nicotinic acetylcholine receptors in lung inflammation and carcinogenesis: friends or foes? J. Cell. Physiol. 234, 14666–14679. doi: 10.1002/jcp.28220 30701535

[ref21] Hansen M. V. Rosenberg J. Gögenur I. (2013). Lack of circadian variation and reduction of heart rate variability in women with breast cancer undergoing lumpectomy: a descriptive study. Breast Cancer Res. Treat. 140, 317–322. doi: 10.1007/s10549-013-2631-x, PMID: 23860927

[ref22] Hart P. C. Rajab I. M. Alebraheem M. Potempa L. A. (2020). C-reactive protein and cancer-diagnostic and therapeutic insights. Front. Immunol. 11:595835. doi: 10.3389/fimmu.2020.595835, PMID: 33324413 PMC7727277

[ref23] Hoca A. Yildiz M. Ozyigit G. (2012). Evaluation of the effects of mediastinal radiation therapy on autonomic nervous system. Med. Oncol. 29, 3581–3586. doi: 10.1007/s12032-012-0237-5, PMID: 22528518

[ref24] Hu S. Lou J. Zhang Y. Chen P. (2018). Low heart rate variability relates to the progression of gastric cancer. World J. Surg. Oncol. 16:49. doi: 10.1186/s12957-018-1348-z, PMID: 29514707 PMC5842632

[ref25] Huan H. B. Wen X. D. Chen X. J. Wu L. Wu L. L. Zhang L.. (2017). Sympathetic nervous system promotes hepatocarcinogenesis by modulating inflammation through activation of alpha1-adrenergic receptors of Kupffer cells. Brain Behav. Immun. 59, 118–134. doi: 10.1016/j.bbi.2016.08.016, PMID: 27585737

[ref26] Kim K. Chae J. Lee S. (2015). The role of heart rate variability in advanced non-small-cell lung cancer patients. J. Palliat. Care 31, 103–108. doi: 10.1177/082585971503100206, PMID: 26201212

[ref27] Kloter E. Barrueto K. Klein S. D. Scholkmann F. Wolf U. (2018). Heart rate variability as a prognostic factor for cancer survival - a systematic review. Front. Physiol. 9:623. doi: 10.3389/fphys.2018.00623, PMID: 29896113 PMC5986915

[ref28] Lee G. M. Fattinger S. Mouthon A. L. Noirhomme Q. Huber R. (2013). Electroencephalogram approximate entropy influenced by both age and sleep. Front. Neuroinform. 7:33. doi: 10.3389/fninf.2013.00033, PMID: 24367328 PMC3852001

[ref29] Li G. Wu S. Zhao H. Guan W. Zhou Y. Shi B. (2022). Non-invasive prognostic biomarker of lung cancer patients with brain metastases: recurrence quantification analysis of heart rate variability. Front. Physiol. 13:987835. doi: 10.3389/fphys.2022.987835, PMID: 36148296 PMC9486009

[ref30] Lombardi F. Stein P. K. (2011). Origin of heart rate variability and turbulence: an appraisal of autonomic modulation of cardiovascular function. Front. Physiol. 2:95. doi: 10.3389/fphys.2011.00095, PMID: 22163222 PMC3233900

[ref31] Marwan N. Carmenromano M. Thiel M. Kurths J. (2007). Recurrence plots for the analysis of complex systems. Phys. Rep. 438, 237–329. doi: 10.1016/j.physrep.2006.11.001

[ref32] Marwan N. Wessel N. Meyerfeldt U. Schirdewan A. Kurths J. (2002). Recurrence-plot-based measures of complexity and their application to heart-ratevariability data. Phys. Rev. E Stat. Nonlinear Soft Matter Phys. 66:026702. doi: 10.1103/PhysRevE.66.026702, PMID: 12241313

[ref33] Mohebbi M. Ghassemian H. (2011). Prediction of paroxysmal atrial fibrillation using recurrence plot-based features of the RR-interval signal. Physiol. Meas. 32, 1147–1162. doi: 10.1088/0967-3334/32/8/010, PMID: 21709338

[ref34] Mohseni M. Redies C. Gast V. (2022). Approximate entropy in canonical and non-canonical fiction. Entropy 24:278. doi: 10.3390/e24020278, PMID: 35205572 PMC8870941

[ref35] Moody G. Mark R. Zoccola A. Mantero S. (1985). Derivation of respiratory signals from multi-lead ECGs. Comput. Cardiol. 12, 113–116.

[ref36] Mouton C. Ronson A. Razavi D. Delhaye F. Kupper N. Paesmans M.. (2012). The relationship between heart rate variability and time-course of carcinoembryonic antigen in colorectal cancer. Auton. Neurosci. 166, 96–99. doi: 10.1016/j.autneu.2011.10.002, PMID: 22070982

[ref37] Niskanen J. P. Tarvainen M. P. Ranta-Aho P. O. Karjalainen P. A. (2004). Software for advanced HRV analysis. Comput. Methods Prog. Biomed. 76, 73–81. doi: 10.1016/j.cmpb.2004.03.004, PMID: 15313543

[ref38] Pan J. Tompkins W. J. (1985). A real-time QRS detection algorithm. IEEE Trans. Biomed. Eng. 32, 230–236. doi: 10.1109/TBME.1985.325532 3997178

[ref39] Peng Y. Sun Z. (2011). Characterization of QT and RR interval series during acute myocardial ischemia by means of recurrence quantification analysis. Med. Biol. Eng. Comput. 49, 25–31. doi: 10.1007/s11517-010-0671-5, PMID: 20725861

[ref40] Saku K. Kishi T. Sakamoto K. Hosokawa K. Sakamoto T. Murayama Y.. (2014). Afferent vagal nerve stimulation resets baroreflex neural arc and inhibits sympathetic nerve activity. Phys. Rep. 2:e12136. doi: 10.14814/phy2.12136, PMID: 25194023 PMC4270242

[ref41] Schwartz P. J. De Ferrari G. M. (2011). Sympathetic-parasympathetic interaction in health and disease: abnormalities and relevance in heart failure. Heart Fail. Rev. 16, 101–107. doi: 10.1007/s10741-010-9179-1, PMID: 20577900

[ref42] Shi B. Wang L. Yan C. Chen D. Liu M. Li P. (2019). Nonlinear heart rate variability biomarkers for gastric cancer severity: a pilot study. Sci. Rep. 9:13833. doi: 10.1038/s41598-019-50358-y, PMID: 31554856 PMC6761171

[ref43] Sloan E. K. Priceman S. J. Cox B. F. Yu S. Pimentel M. A. Tangkanangnukul V.. (2010). The sympathetic nervous system induces a metastatic switch in primary breast cancer. Cancer Res. 70, 7042–7052. doi: 10.1158/0008-5472.CAN-10-0522, PMID: 20823155 PMC2940980

[ref44] Stachowiak P. Milchert-Leszczyńska M. Falco M. Wojtarowicz A. Kaliszczak R. Safranow K.. (2018). Heart rate variability during and after chemotherapy with anthracycline in patients with breast cancer. Kardiol. Pol. 76, 914–916. doi: 10.5603/KP.2018.0098, PMID: 29756192

[ref45] Sun R. Wang Y. (2008). Predicting termination of atrial fibrillation based on the structure and quantification of the recurrence plot. Med. Eng. Phys. 30, 1105–1111. doi: 10.1016/j.medengphy.2008.01.008, PMID: 18343707

[ref46] Sung H. Ferlay J. Siegel R. L. Laversanne M. Soerjomataram I. Jemal A.. (2021). Global cancer statistics 2020: GLOBOCAN estimates of incidence and mortality worldwide for 36 cancers in 185 countries. CA Cancer J. Clin. 71, 209–249. doi: 10.3322/caac.21660, PMID: 33538338

[ref47] Taniguchi K. Karin M. (2014). IL-6 and related cytokines as the critical lynchpins between inflammation and cancer. Semin. Immunol. 26, 54–74. doi: 10.1016/j.smim.2014.01.001, PMID: 24552665

[ref48] Tracey K. J. (2009). Reflex control of immunity. Nat. Rev. Immunol. 9, 418–428. doi: 10.1038/nri2566, PMID: 19461672 PMC4535331

[ref49] Vaillancourt D. E. Newell K. M. (2002). Changing complexity in human behavior and physiology through aging and disease. Neurobiol. Aging 23, 1–11. doi: 10.1016/s0197-4580(01)00247-0, PMID: 11755010

[ref50] Vanderlei L. C. Pastre C. M. Hoshi R. A. Carvalho T. D. Godoy M. F. (2009). Basic notions of heart rate variability and its clinical applicability. Rev. Bras. Cir. Cardiovasc. 24, 205–217. doi: 10.1590/s0102-76382009000200018, PMID: 19768301

[ref51] Voss A. Schulz S. Schroeder R. Baumert M. Caminal P. (2009). Methods derived from nonlinear dynamics for analysing heart rate variability. Philos. Trans. A. Math. Phys. Eng. Sci. 367, 277–296. doi: 10.1098/rsta.2008.0232, PMID: 18977726

[ref52] Wang Y. M. Cheng J. Y. Wang C. J. Hseu S. S. Huang E. Y. (2021). Outcomes and prognosis of non-elderly patients with brain metastases-a prospective cohort incorporating individualized assessment of heart rate variability. J. Pers. Med. 11:1049. doi: 10.3390/jpm11111049, PMID: 34834401 PMC8618592

[ref53] Wang H. M. Liao Z. X. Komaki R. Welsh J. W. O'Reilly M. S. Chang J. Y.. (2013). Improved survival outcomes with the incidental use of beta-blockers among patients with non-small-cell lung cancer treated with definitive radiation therapy. Ann. Oncol. 24, 1312–1319. doi: 10.1093/annonc/mds616, PMID: 23300016 PMC3629895

[ref54] Wang Y. M. Wu H. T. Huang E. Y. Kou Y. R. Hseu S. S. (2013). Heart rate variability is associated with survival in patients with brain metastasis: a preliminary report. Biomed. Res. Int. 2013:503421. doi: 10.1155/2013/503421, PMID: 24102056 PMC3786541

[ref55] Webber C. L. Jr. Zbilut J. P. (1994). Dynamical assessment of physiological systems and states using recurrence plot strategies. J. Appl. Physiol. 1985, 965–973. doi: 10.1152/jappl.1994.76.2.965 8175612

[ref56] Williams D. P. Koenig J. Carnevali L. Sgoifo A. Jarczok M. N. Sternberg E. M.. (2019). Heart rate variability and inflammation: a meta-analysis of human studies. Brain Behav. Immun. 80, 219–226. doi: 10.1016/j.bbi.2019.03.009, PMID: 30872091

[ref57] Wu S. Chen M. Wang J. Shi B. Zhou Y. (2021). Association of Short-Term Heart Rate Variability with Breast Tumor Stage. Front. Physiol. 12:678428. doi: 10.3389/fphys.2021.678428, PMID: 34566672 PMC8461241

[ref58] Wu S. Li G. Guan W. Zhao H. Wang J. Zhou Y.. (2022). Low heart rate variability predicts poor overall survival of lung cancer patients with brain metastases. Front. Neurosci. 16:839874. doi: 10.3389/fnins.2022.839874, PMID: 35250470 PMC8891474

[ref59] Yentes J. M. Hunt N. Schmid K. K. Kaipust J. P. McGrath D. Stergiou N. (2013). The appropriate use of approximate entropy and sample entropy with short data sets. Ann. Biomed. Eng. 41, 349–365. doi: 10.1007/s10439-012-0668-3, PMID: 23064819 PMC6549512

[ref60] Zheng M. (2016). Classification and pathology of lung cancer. Surg. Oncol. Clin. N. Am. 25, 447–468. doi: 10.1016/j.soc.2016.02.003 27261908

[ref61] Zimatore G. Garilli G. Poscolieri M. Rafanelli C. Terenzio Gizzi F. Lazzari M. (2017). The remarkable coherence between two Italian far away recording stations points to a role of acoustic emissions from crustal rocks for earthquake analysis. Chaos 27:043101. doi: 10.1063/1.4979351, PMID: 28456156

